# Splenectomy in Thalassemia: The Role of Surgery as an Adjunct to Medical Management

**DOI:** 10.7759/cureus.62834

**Published:** 2024-06-21

**Authors:** Shishir Kumar, Shivraj Chauhan

**Affiliations:** 1 Department of Surgery, Tata Main Hospital, Jamshedpur, IND; 2 Department of General Surgery, Tata Main Hospital, Jamshedpur, IND

**Keywords:** pulmonary hypertension, chelation, hypersplenism, thalassemia, splenectomy

## Abstract

Introduction

Beta thalassemia is a hemoglobinopathy characterized by defective production of the beta chain of hemoglobin, leading to irreversible destruction of RBCs, splenomegaly, pancytopenia, and a requirement for multiple transfusions. This condition necessitates iron chelation therapy, and splenectomy is often performed to manage hypersplenism.

Methods

This report includes a series of seven diagnosed cases of beta thalassemia with hypersplenism, all of whom underwent open splenectomy. Preoperative transfusions were administered to achieve target hemoglobin and platelet counts of 9 g/dL and 50,000/µL, respectively.

Results

The study included seven patients diagnosed with beta thalassemia, all of whom underwent open splenectomy. Among these, three patients also had concomitant cholecystectomy due to the presence of gallstones. The primary indication for performing splenectomy was hypersplenism. Preoperative transfusions were administered to ensure target hemoglobin levels of 9 g/dL and platelet counts of 50,000/µL. All patients were successfully discharged with minimal morbidity and no reported mortality. The longest follow-up period observed in this series was 10 months post-splenectomy, which limited the assessment of long-term effects.

Conclusion

Open splenectomy for hypersplenism in patients with beta thalassemia appears to be a safe and effective procedure with minimal short-term morbidity and no mortality observed in this series. However, due to the limited follow-up duration, the long-term effects of splenectomy in these patients could not be evaluated. Further studies with longer follow-up are needed to assess the long-term outcomes of splenectomy in beta thalassemia patients.

## Introduction

The indications for splenectomy are well established in medical literature. Given the spleen's crucial role in the body's immune mechanisms, there has been a gradual shift towards preserving the spleen, even in cases of severe (grade IV) splenic trauma [1]. In the context of hemolytic anemia, the spleen is pivotal in the removal of deformed RBCs from circulation through processes known as pitting and culling, which leads to an enlargement of the spleen [2]. This enlargement, when coupled with cytopenia (a reduction in the number of blood cells due to their entrapment in the enlarged spleen) and pain, forms a condition known as hypersplenism. Hypersplenism is a significant therapeutic indication for splenectomy [3].

The removal of the spleen in such scenarios not only alleviates the symptoms of hypersplenism but also decreases the need for frequent blood transfusions, thereby mitigating the risk of iron overload.

In our clinical practice, we have managed seven cases of splenectomy in patients with beta thalassemia, all of whom presented with hypersplenism. Post-splenectomy, these patients experienced a resolution of hypersplenism symptoms and a significant reduction in the necessity for multiple blood transfusions. Despite the splenectomy, iron chelation therapy was continued in all patients to manage the iron overload condition effectively. This comprehensive approach highlights the dual benefits of splenectomy in alleviating hypersplenism and reducing transfusion dependency, while ongoing iron chelation addresses the associated iron overload.

## Materials and methods

This case series examines seven patients who underwent splenectomy for hypersplenism at a tertiary care industrial hospital in Jamshedpur, Jharkhand, over a study period from December 2022 to November 2023. Institutional ethical committee approval was obtained for the study (TMH/IEC/MAY/150/2024). All patients, ranging in age from 5 to 19 years with an average age of 12.8 years, were diagnosed with beta thalassemia accompanied by hypersplenism.

As part of the preoperative preparations, all patients received vaccinations one month prior to surgery, including the Pentavalent vaccine (covering Diphtheria, Pertussis, Tetanus, Hepatitis B, and Haemophilus influenzae type B), as well as the pneumococcal and meningococcal vaccines. Comprehensive preoperative blood work was conducted, and anesthetic fitness was confirmed. Additionally, preoperative transfusions with appropriate blood products were administered to achieve target hemoglobin and platelet counts of 9 g/dL and 50,000/µL, respectively. Routine ultrasonogram screening for gallstones was also performed.

The clinical and diagnostic data of seven patients with beta thalassemia and hypersplenism who underwent splenectomy are summarized in Table 1. The hemoglobin levels of these patients ranged from 4.3 to 7.2 g/dL, all of which are below the normal range of 11.5-16.5 g/dL, indicating varying degrees of anemia. Their total leukocyte counts (TLC) were also low, ranging from 2,000 to 3,600 per µL, compared to the normal range of 4,000-11,000 per µL, reflecting leukopenia. Platelet counts were significantly reduced, with values between 26,000 and 54,000 per µL, well below the normal range of 150,000-410,000 per µL, indicating thrombocytopenia.

**Table 1:** Pre-operative blood parameters and average number of blood transfusions. Hb: Hemoglobin; cm: Centimeter; S. No: Serial number. Hb Reference Range: 11.5-16.5 gram per deciliter. TLC Reference Range: 4,000-11,000 per cubic millimeter. Platelet Reference Range: 4.5-5.5 million per cubic millimeter.

S. No	Hb (g/dL)	Total leukocyte count (TLC)	Platelet counts (x1000/µL)	Gall stones	Pre-operative vaccination	Splenic span (along long axis)	Transfusion/month
1	6.4	3200	42	Yes	Yes	28 cm	3-4
2	5.2	2400	54	No	Yes	30 cm	2-3
3	5.0	2700	38	No	Yes	26 cm	3-4
4	4.3	2800	26	Yes	Yes	24 cm	3-4
5	6.4	3400	34	No	Yes	29 cm	2-3
6	7.0	3600	32	No	Yes	22 cm	3-4
7	7.2	2000	28	Yes	Yes	28 cm	3-4

Gallstones were detected in three patients (patients 1, 4, and 7), while the remaining four patients did not have gallstones. All patients received preoperative vaccinations. The splenic span, measured along the long axis, ranged from 22 cm to 30 cm, confirming splenomegaly in all cases. The frequency of blood transfusions required per month varied from 2 to 4 transfusions among the patients. This data highlights the severity of their conditions, marked by anemia, leukopenia, thrombocytopenia, and significant splenomegaly, necessitating frequent blood transfusions. Preoperative vaccinations were consistently administered, and nearly half of the patients had gallstones.

**Figure 1:**
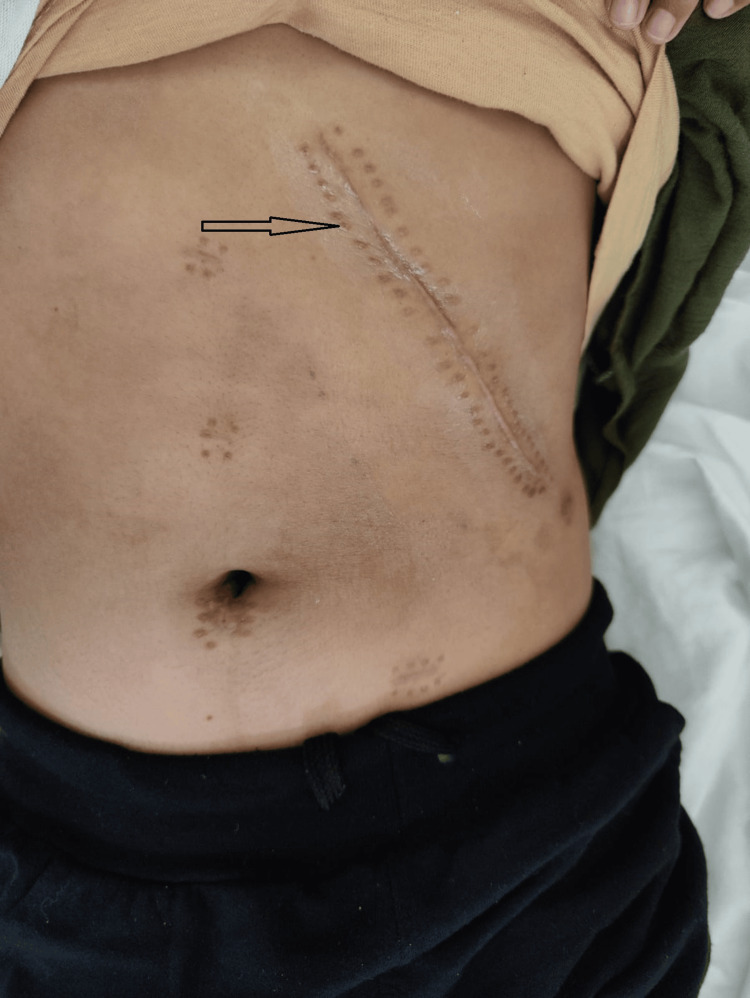
Left subcostal incision, marked by an asterisk. The figure depicts the healed subcostal incision line taken at the follow-up visit of the patient.

All patients underwent open splenectomy by a single surgeon. A left subcostal incision was made in five patients (Figure 1). A midline laparotomy approach was taken in three patients for concomitant cholecystectomy.

**Figure 2:**
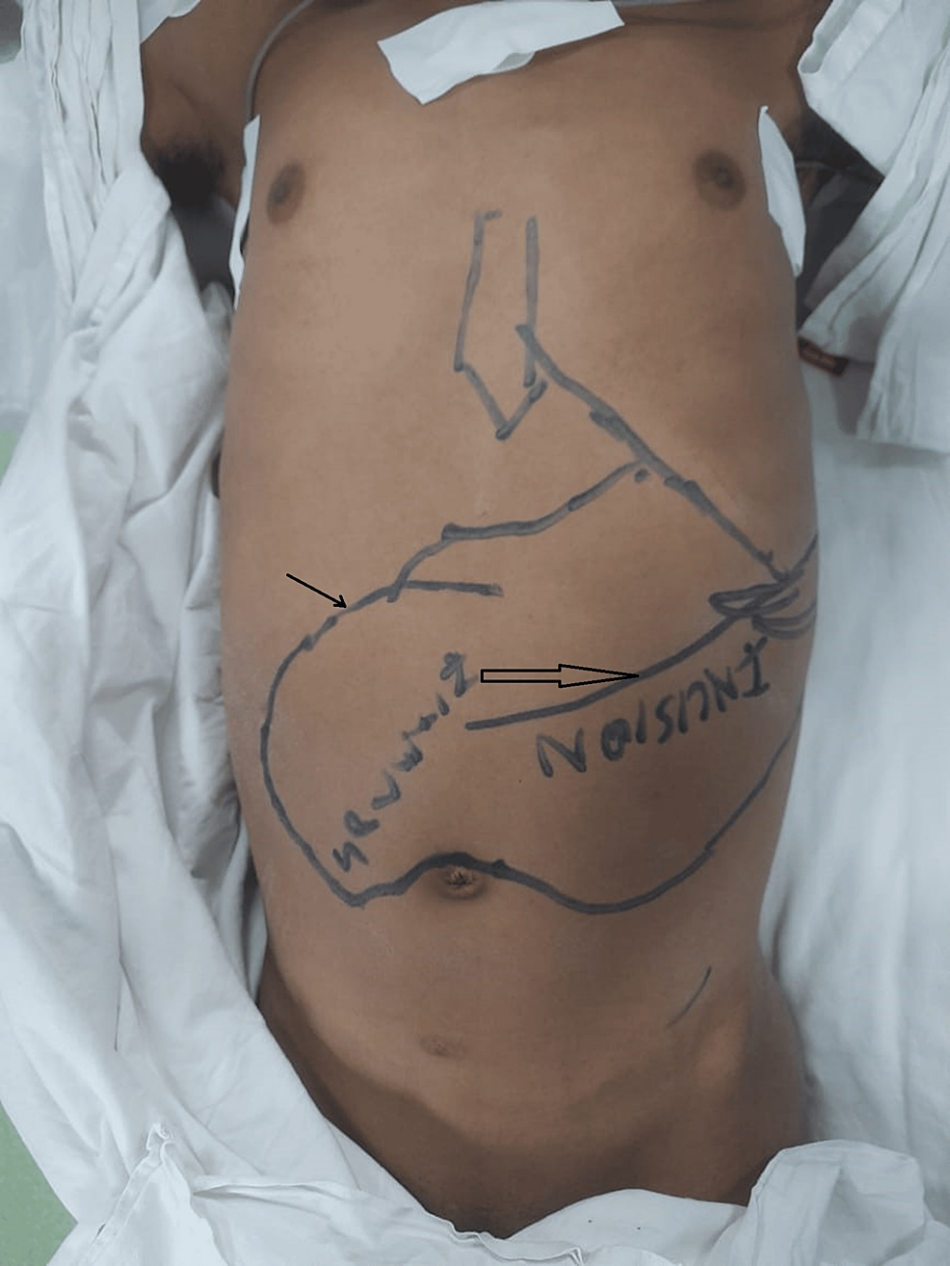
Left thoracoabdominal incision, marked by an asterisk. The figure depicts the abdominal part of the thoracoabdominal incision (hollow asterisk). The splenic border is marked by a horizontal arrow.

In n-1 patient, a left thoracoabdominal incision was made for massive enlargement of the spleen. The thoracic incision extended from the seventh intercostal space to the posterior axillary line. This extension was needed to address the enlarged veins at the upper pole of the spleen and enlarged diaphragmatic collaterals in this case to ensure secure hemostasis (Figure 2).

**Figure 3:**
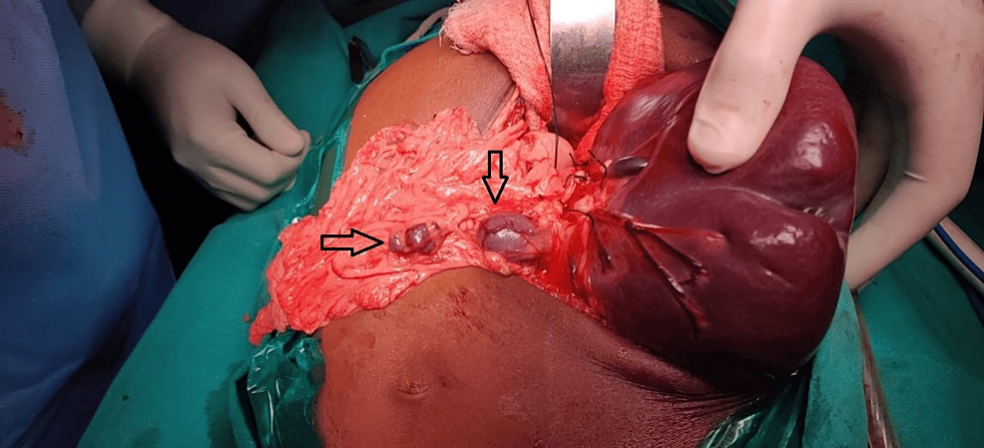
Spleniculi, shown by an asterisk. The figure depicts two spleniculi dissected during the splenectomy.

The standard procedure of first ligating the splenic artery, followed by the ligation of the splenic vein, was meticulously performed. Single-donor platelet transfusions were initiated immediately after the ligation of the splenic artery. An intra-abdominal drain was placed in the splenic bed for all patients, and the abdominal wall was subsequently closed. For the patient who underwent a thoracoabdominal incision, an intercostal tube was inserted. Additionally, a thorough intraoperative search for accessory spleens (spleniculi) was conducted in all cases, ensuring the meticulous removal of any spleniculi found (Figure 3).

All patients were allowed to consume clear oral fluids by the first postoperative day, with full oral feeds permitted by the second postoperative day. The average drainage output was 100 ml on the first postoperative day and decreased to 30 ml by the second postoperative day. The abdominal drain was removed by the fourth postoperative day for all patients. Full feeds were achieved within 48 hours (by the second postoperative day). No instances of surgical site infections or lung complications were observed in this series. The average length of hospital stay was 5 days. Liver biopsy specimens from all patients revealed hemosiderosis. The shortest and longest follow-up periods in this series were 1 month and 10 months, respectively.

## Results

The benefits of splenectomy in these patients were measured by increases in hemoglobin, platelet counts, and decreases in the number of blood transfusions, as compared to pre-splenectomy states. The mean Hb at presentation was 5.92 g/dL. Mean Hb at the first and third postoperative months were 9.7 g/dL and 9.6 g/dL, respectively. Mean platelet counts showed a marked improvement from 36,000/µL at presentation to 104,400/µL at the first postoperative month and 116,000/µL at the third postoperative month. All of them showed marked resolution of abdominal symptoms (increased appetite, abdominal pain) and decreased transfusion requirements (from an average of 1-2 transfusions per week to 1-2 every three months). Also, splenectomy in these cases eliminated the risk of splenic rupture. As a part of the standard treatment protocol, iron chelation therapy in the form of Deferoxamine was continued even in the postoperative period.

At baseline, patients presented with a mean hemoglobin level of 5.92 g/dL, indicative of severe anemia. Post-splenectomy, there was a remarkable rise in hemoglobin levels, with mean values reaching 9.7 g/dL and 9.6 g/dL at the first and third postoperative months, respectively. This substantial improvement in hemoglobin levels signifies effective management of anemia following splenectomy (Table 2).

**Table 2:** Postoperative blood parameters and number of blood transfusions. OPSI: Overwhelming post splenectomy infection; Hb: Hemoglobin; Kg: Kilogram; g/dl: Gram per deciliter; Cu.mm: Cubic millimeter. Hb Reference Range: 11.5-16.5 gram per deciliter TLC Reference Range: 4,000-11,000 per cubic millimeter Platelet Reference Range: 4.5-5.5 million per cubic millimeter

S. No.	Hb at 1^st^ postoperative month. (g/dl)	Hb at 3^rd^ postoperative month	Platelet counts (x1000/µL) (1^st ^and 3^rd^ postoperative months)	Weight of excised spleen (kg)	OPSI	Transfusion/3-months
1	9.2	9.0	100/110	2.5	Nil	1
2	10.4	9.8	105/120	2.2	Nil	1-2
3	8.4	9.0	96/110	1.8	Nil	1
4	9.4	9.6	110/110	2.6	Nil	1
5	11.2	10.4	106/116	1.9	Nil	1
6	9.6	9.8	104/130	1.7	Nil	2/3 months
7	10.2	-	110/-	3.2	Nil	-(follow up for 01 month)

Furthermore, platelet counts demonstrated a significant enhancement post-splenectomy, escalating from an average of 36,000/µL at presentation to 104,400/µL at the first postoperative month and 116,000/µL at the third postoperative month. This notable increase in platelet counts reflects the successful resolution of thrombocytopenia, reducing the risk of bleeding complications and improving overall hematologic function (Table 2).

In addition to hematologic improvements, all patients experienced resolution of abdominal symptoms, including increased appetite and relief from abdominal pain, following splenectomy. Moreover, there was a substantial reduction in the need for blood transfusions, with the frequency decreasing from an average of 1-2 transfusions per week to 1-2 every three months. This decrease in transfusion requirements highlights the efficacy of splenectomy in reducing dependence on external blood support and improving patient outcomes.

Notably, splenectomy effectively eliminated the risk of splenic rupture in these patients, providing long-term safety and preventing potential life-threatening complications. The continuation of iron chelation therapy with Deferoxamine postoperatively further contributed to the management of iron overload, ensuring optimal hematologic outcomes and reducing the risk of associated complications.

Moreover, no signs suggestive of overwhelming post-splenectomy infections (OPSI) were observed during the follow-up period, underscoring the safety of splenectomy in these patients. All patients received triple vaccination one month prior to splenectomy, further minimizing the risk of postoperative infections. Additionally, thrombocytosis or visceral thromboembolism was not reported in any patient during the postoperative period up to the last follow-up, emphasizing the absence of significant thrombotic complications post-splenectomy.

The detailed postoperative blood parameters and transfusion requirements for each patient at the first and third postoperative months are summarized in Table 2.

Table 3 provides insights into the percentage increase in hemoglobin and platelet counts at the first and third months post-splenectomy, further corroborating the favorable outcomes observed in this study.

**Table 3:** Percentage increase in hemoglobin and platelet counts at the first and third months post-splenectomy. %: Percentage; Hb: Hemoglobin.

Blood parameter	% increase at first month	% increase at third month
Hb	63.8	62.2
Platelets	190	222

These findings collectively underscore the beneficial effects of splenectomy in patients with beta thalassemia and hypersplenism, highlighting its role in improving hematologic parameters, reducing transfusion requirements, and enhancing overall clinical outcomes.

## Discussion

Splenectomy has well established indications in surgical literature, as part of surgical intervention for indications like trauma, malignancy, splenic abscess etc [4,5]. With increasing evidence of central role of spleen in immunological functions in body, the emphasis is more on splenic conservation, rather than splenectomy. The maneuvers like splenic embolization for splenic artery aneurysm, and partial splenectomy techniques are testament to splenic role in body’s immune defense mechanism [6,7]. One of the standard indications for splenectomy is in hemolytic anemia. Sequestration crises in sickle cell anemia and the need for multiple blood transfusions in thalassemia are the main indicators for elective splenectomy in such cases [8]. The underlying indication being hypersplenism, characterized by a triad of massive splenomegaly, leukopenia, and thrombocytopenia. Multiple transfusions necessitate repeated hospital admissions and ultimately lead to iron deposition (hemosiderosis) and its resultant sequelae [9]. The role of splenectomy, as in this case series, is to reduce the number of blood transfusions, and thus iron overload states. In our series, the average number of blood transfusions showed a reduction from an average of three transfusions per week to an average of one transfusion every two months. Though to assess the sustainability of this effect, a much longer period of follow-up is necessary, as literature suggests that this reduction in frequency of blood transfusions is not sustained in the long term [10]. The most feared postoperative complication of splenectomy is OPSI [11]. With the standard recommended guidelines of triple vaccination in both emergency and elective splenectomies, the overall rate of OPSI has decreased to 0.5-1% overall [12]. In our series, all cases were preoperatively vaccinated one month prior to splenectomy. In the short follow-up period also, none of the cases showed any signs suggestive of OPSI. Annual administration of influenza vaccines, and oral amoxicillin/clavulanic acid at the onset of a febrile illness are established measures for prevention of OPSI in splenectomized patients [13]. Thrombocytosis has been documented in 75-85% of patients undergoing splenectomy. The rate of visceral thromboembolism is reported as 5% [14]. In our series, mean platelet counts after the 1st and 3rd postoperative months were 104,000/µL and 116,000/µL, respectively. Administration of low-dose aspirin has been documented to be beneficial in patients in whom the thrombocytosis exceeds a count of 7 lac/µL [15]. Pulmonary hypertension has been reported as a long-standing complication of splenectomy in cases of hemolytic anemia [16]. The underlying mechanism is multifactorial, and the associated hypercoagulability in patients with hematological disorders is described to be responsible for this complication. As per the published literature, new-onset pulmonary hypertension in such cases takes 2-35 years to develop [17]. This effect could not be studied in our series as the maximum follow-up in our series has been of the duration of 10 months only. The surgical approach to splenectomy, viz open or laparoscopic, is a surgeon’s perspective, rather than an absolute one. In our series, all patients underwent splenectomy by open method. The most common incision used was the left subcostal. A midline incision was used in patients needing concomitant cholecystectomy, and a thoraco-abdominal incision in a patient with massive enlargement of the spleen. Laparoscopic surgery in such cases has its own set of challenges due to underlying coagulopathy, thrombocytopenia, and large-sized spleen. Surgical literature shows that in such cases, the need for a hand-assist port or total conversion to an open procedure facilitates the completion of the procedure. The incidence of intraoperative complications has been reported to be higher in laparoscopic splenectomies [18]. Also, a higher American Society of Anaesthesiologists (ASA) score and larger spleen size have been reported as independent risk factors for intraoperative complications in elective splenectomies [19]. In our series, the average splenic span of 26.7 cm, and weight of 2.27 kg made open splenectomy a more favorable approach.

Limitations

This article considers only seven cases operated over a time span of 12 months, the longest follow-up period being only 10 months. The long-term effects of splenectomy, like pulmonary hypertension, could not be assessed in such a short follow-up period. Also, due to the small set of patients, the role of splenectomy in other hemolytic states like sickle cell anemia could not be studied. The authors intend to keep the patients on regular follow-up to assess the long-term effects of splenectomy in these patients.

## Conclusions

Splenectomy plays a crucial role in the comprehensive management of thalassemia, a fact well acknowledged within the medical community. While the primary management pillars typically revolve around timely blood transfusions and iron chelation therapy, the significance of splenectomy, whether performed through open or minimally invasive techniques, cannot be overlooked, especially in patients grappling with hypersplenism and recurrent blood transfusions, along with their attendant complications. Iron overload resulting from frequent transfusions necessitates meticulous chelation therapy, which forms the cornerstone of management to mitigate associated risks.

Regular monitoring through follow-up hospital visits remains imperative, serving as a proactive measure to detect and address potential complications such as pulmonary hypertension. Adherence to well-established protocols concerning preoperative vaccination further mitigates the morbidity associated with splenectomy in such cases. The holistic approach to thalassemia management underscores the importance of splenectomy as a strategic intervention in alleviating the burden of hypersplenism and its sequelae, thereby improving patient outcomes and quality of life.
